# Prevalence, aetiologies and prognosis of the symptom cough in primary care: a systematic review and meta-analysis

**DOI:** 10.1186/s12875-021-01501-0

**Published:** 2021-07-12

**Authors:** Milena Bergmann, Jörg Haasenritter, Dominik Beidatsch, Sonja Schwarm, Kaja Hörner, Stefan Bösner, Paula Grevenrath, Laura Schmidt, Annika Viniol, Norbert Donner-Banzhoff, Annette Becker

**Affiliations:** Department of General Practice / Family Medicine, University of Marburg, Karl-von-Frisch-Str. 4, 35043 Marburg, Germany

**Keywords:** Cough, General practice, Primary care, Diagnosis, Prevalence, Aetiology, Prognosis, Systematic review, Symptom-evaluating study

## Abstract

**Background:**

Cough is a relevant reason for encounter in primary care. For evidence-based decision making, general practitioners need setting-specific knowledge about prevalences, pre-test probabilities, and prognosis. Accordingly, we performed a systematic review of symptom-evaluating studies evaluating cough as reason for encounter in primary care.

**Methods:**

We conducted a search in MEDLINE and EMBASE. Eligibility criteria and methodological quality were assessed independently by two reviewers. We extracted data on prevalence, aetiologies and prognosis, and estimated the variation across studies. If justifiable in terms of heterogeneity, we performed a meta-analysis.

**Results:**

We identified 21 eligible studies on prevalence, 12 on aetiology, and four on prognosis. Prevalence/incidence estimates were 3.8–4.2%/12.5% (Western primary care) and 10.3–13.8%/6.3–6.5% in Africa, Asia and South America. In Western countries the underlying diagnoses for acute cough or cough of all durations were respiratory tract infections (73–91.9%), influenza (6–15.2%), asthma (3.2–15%), laryngitis/tracheitis (3.6–9%), pneumonia (4.0–4.2%), COPD (0.5–3.3%), heart failure (0.3%), and suspected malignancy (0.2–1.8%). Median time for recovery was 9 to 11 days. Complete recovery was reported by 40.2- 67% of patients after two weeks, and by 79% after four weeks. About 21.1–35% of patients re-consulted; 0–1.3% of acute cough patients were hospitalized, none died. Evidence is missing concerning subacute and chronic cough.

**Conclusion:**

Prevalences and incidences of cough are high and show regional variation. Acute cough, mainly caused by respiratory tract infections, is usually self-limiting (supporting a “wait-and-see” strategy). We have no setting-specific evidence to support current guideline recommendations concerning subacute or chronic cough in Western primary care. Our study presents epidemiological data under non non-pandemic conditions. It will be interesting to compare these data to future research results of the post-pandemic era.

**Supplementary Information:**

The online version contains supplementary material available at 10.1186/s12875-021-01501-0.

## Background

Nearly every person has experienced an episode of cough in their lifetime. Based on population, the prevalence of cough in Europe and the USA is 9–33% [1]. Severe cough can significantly impair health-related quality of life and be linked i.a. to depression, urinary incontinence, syncope, social embarrassment, sleep disturbance and depression [2, 3]. While most episodes of cough are benign and self-limiting, in some cases the symptom points to severe illnesses like pneumonia or lung cancer [4].

General practitioners (GPs) play an important role as gatekeepers. Based on history and examination, they triage self-limiting symptoms and severe, possibly life-limiting diseases and decide about further testing, treatment and referral. To support the clinical decision-making process, GPs need to know the percentage distribution of possible aetiologies in order to correctly interpret the clinical signs. This is different from inpatient settings because patients in family practices, which are the first point of contact, are more likely to have an uncomplicated cause of their cough than are patients in a hospital. Nevertheless, family physicians need to work with the pre-test probabilities of potentially dangerous illnesses in their setting, and also the most likely prognosis of their patients.

Evidence is given by cough guidelines [5–7]. However, data often derives from secondary or tertiary care settings which show different pre-test probabilities. Symptom-evaluating studies in primary care are needed for a more rational and evidence-based approach in setting-specific decision making [8].

Therefore, we performed a systematic review aiming to answer the following research questions: (1) What is the frequency / prevalence of cough in primary care? (2) What are the underlying aetiologies and their frequencies? and (3) What is the prognosis of patients presenting with cough in primary care?

## Methods

### Data sources and search strategy

We conducted a systematic review including all studies evaluating the symptom “cough” as a reason for encounter in primary care. The methods were based on the PRISMA statement [9] and on recommendations for symptom-evaluating studies by Donner-Banzhoff et al. 2001 [8]. The study methods including eligibility criteria and analysis were pre-specified in a protocol. Our research group applied the same methods for the symptoms tiredness, abdominal pain, headache, chest pain, dyspnoea, dizziness, and back pain [10–14].

We performed a systematic search in MEDLINE (2012) and EMBASE (2015), updated 2019 resp. 2020, addressing publications in English, German, and French. A snowball search included the reference lists of all articles and reviews. The search syntax combined the terms “cough” AND “general practice” in various notations OR their MESH terms in title or abstract. Alternatively, we considered papers on “cough” published in journals representing primary care research OR papers in which the term “primary care” appeared in different notations in the affiliation of at least the main author. The entire search syntax can be found in Additional File 1.

### Study selection and data extraction

We screened titles and abstracts and the eligible full text articles with respect to the criteria given in Table 1. Eligible studies focusing solely on children were excluded from data analysis and will be published elsewhere.

**Table 1:** Inclusion and exclusion criteria for screening of titles/abstracts and eligible full text articles

Category	Inclusion criteria	Exclusion criteria	Assessment in
(1) Study design	original quantitative study design regardless of study quality, risk of bias or type of data assessment	qualitative studies, case reports, reviews, full text was not available	titles/abstracts, eligible full text articles
(2) Setting	primary care / general practice	secondary or tertiary care, emergency departments, out-of-hours-services, population-based settings	titles/abstracts, eligible full text articles
(3) Symptom	cough as the primary or secondary reason for the consultation	patients were systematically asked whether they are coughing	titles/abstracts, eligible full text articles
(4) Selection	unselected study population regarding the likelihood of a specific condition as the underlying aetiology	specific groups of cough patients were explicitly included or excluded (e.g. cough due to respiratory tract infections, a mandatory combination of cough with another symptom or an exclusion of patients with underlying conditions like asthma or COPD)	eligible full text articles
(5) Outcomes	data on incidence, prevalence, aetiology or the prognosis of cough	no data on incidence, prevalence, aetiology or the prognosis of cough	eligible full text articles

All steps of the selection process (except its update in 2019/2020) were performed and documented by two reviewers (MB, DB/SS) working independently. In case of disagreement, the full text evaluation was revised, inclusion criteria were discussed, and, if necessary, an expert (AB) was consulted.

We extracted bibliographic data (author, publication year, title, journal), country, inclusion criteria, definition of cough, characteristics of physicians and practices, study design, sample size and study duration. For outcomes we extracted data concerning prevalence/incidence, underlying aetiologies and the prognosis of cough. Seven study authors were contacted to complement published data. In case of multiple publications, we extracted data from all eligible reports.

### Assessment of risk of bias

Due to lack of standardized guidelines for assessing risk of bias in symptom-evaluating studies, we followed the criteria published by Donner-Banzhoff et al. [8], which entail four domains with pre-specified key questions related to the potential of bias. Domain A and B refer to all studies dealing with the selection of patients and physicians (description of symptom, inclusion criteria, recruitment, multicentricity), data collection, and patient flow (study design, dropouts). Domain C refers to the aetiological outcomes (the definition of aetiological categories, diagnostic workup). Domain D assesses the quality of the prognostic data (definition of the outcome, inclusion of a comparison group, prognostic workup). Again, two reviewers (MB, KH), working independently, assessed the risk of bias.

### Data analysis

We calculated proportions (with a confidence interval of 95%) on prevalence/incidence data and the underlying aetiologies. If sensible, a meta-analysis was performed. To visualize probability estimates and between-study variation of our data, we used forest plots. To ensure comparability, we grouped studies according to the estimates’ denominators, the duration of cough (both pre-specified) and regional characteristics (post hoc).

For meta-analysis we used the random effects model (assuming a distribution of effects across studies) to weigh estimates of studies in proportion to their significance [15].

Outcomes vary due to differences in study design and bias (methodological heterogeneity) as well as in study population, inclusion criteria, healthcare system and diagnostic workup (clinical heterogeneity) [15]. To quantify heterogeneity, we used χ^2^, p-value, and I^2^. A high χ^2^ and a low p-value correlate with a heterogeneity beyond chance; I^2^ describes the portion of variability that is not due to chance [15].

There were only a few heterogeneous studies providing evidence of prognosis for cough. Therefore these results were analyzed descriptively.

For statistical analysis we used the software R (R Foundation for statistical Computing, Vienna, Austria, version 3.4.4) and RStudio V (RStudio, Inc., version 1.1.442).

## Results

### Search results and study selection

**Fig. 1:**
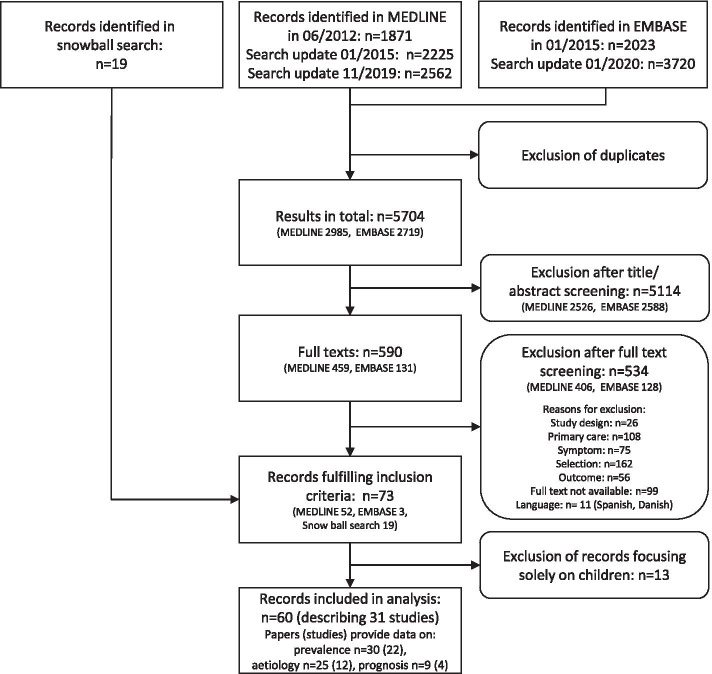
Flowchart selection process

We identified 2,985 references in MEDLINE, 2,719 additional references in EMBASE, and 19 by snowball searching. Screening of titles/abstracts and full texts identified 73 eligible references, of which 60 publications (31 studies) reported data on adults or on patients of all age groups. Of these, 22 provided data on prevalence of cough in primary care, 12 on aetiology and 4 on prognosis. Further details are presented in Fig. 1.

### Included studies

Most studies were conducted in Western countries: In Europe (*n* = 12), in North America (*n* = 6), in both Europe and North America (*n* = 2), and in Australia (*n* = 1). Five studies collected data in Asia, four in Africa, and one in South America, Africa and Asia. Time of publication varied between 1969 and 2018. Studies included 32 to 158,863 patients, 121 to 337,348 consultations, and 385 to 284,348 reasons for encounters. Forty-two per cent to 75% of study populations were women; the overall age ranged from 0 to 103 years (the mean age was 24 to 50 years). One study recruited only patients 65 years and above. Except for one, the study population was recruited prospectively. Further details on study characteristics are presented in Table 2.

**Table 2:** Characteristics of the included studies

Study	Country	Setting	Time of recruite-ment	Data assessment	Study population: number female	Age in sample (years)	Inclusion (IN) / Exclusion (EX) criteria	Out-come
Ajmi 2011 [16]	Tunisia	86 primary health care centres	06/2002–05/2003	prospectively	16,271 consultations 24,882 RFE ♀ 62%	0–103 Ø 24	IN: medical records randomly selected	pre
Albert 2011 [17]	USA	Internet-based medical visits on the University of Pittsburgh Medical Center HealthTrak e-Visit system, users receiving care from a large family medicine practice	08–11/2009	prospectively	121 e-visits ♀ 71%	18–60 +	IN: adult users of an e-visit-system	pre
BEACH Program [18]	Australia	965 randomly selected GPs	04/2015–03/2016	prospectively	97,398 consultations 149,084 RFE ♀ 57%	0–75 +	IN: doctor-patient encounters of all types	pre
Ben Abdelaziz 2004 [19]	Tunisia	6 primary healthcare facilities in the Tunisian Sahel (Sousse)	02/2000–01/2001	prospectively	4022 consultations 6576 RFE ♀ 66,6%	0–100 + Ø 27	IN: all patient-doctor encounters in a randomly chosen 30-day period	pre
Coenen 2004 [20]	Belgium	85 Flemish GPs	02–04/2000 and 02–04/2001	prospectively	810 patients (514 after follow-up) ♀ 57%	Ø 40.9	IN: immunocompetent patients, 18–65 years, new or worsening coughing less than 30 days as (one of) the most important complaint(s) and reason for first encounter	prog
CONTENT Project [21, 22]	Germany	17 general practices in 4 federal states resp. 1 rural out of hours-care centre with 41 GPs	04/2005– 12/2006 resp. 07/2008–06/2011	prospectively	42,469 patients 27,871 RFE resp. 9542 patients 15,886 consultations ♀ 59,7–66%	0–104 Ø 42–48.6	IN: (main) RFE were coded	pre aet
French 2005 [23]	USA	1 walk-in primary care clinic of an academic, tertiary care medical centre	n.r.	prospectively	62 patients ♀ 51,6%	19–88 ♀ Ø 42 ♂ Ø 48	IN: cough < 3 weeks duration EX: none	aet
GRACE Study [24–34]	Belgium, France, Germany, Italy, Netherlands, Poland, Spain, Slovakia, Slovenia, Sweden, UK	294 – 387 GPs in 125 general practices from 16 primary care networks	10/2007–04/2010 resp. 10/2006–03/2007	prospectively	1801 – 3368 patients ♀60–70%	18–61 + Ø45-50	IN: ≥ 18 years, acute or worsened cough (≤ 28 days duration) as main/dominant symptom, or suggested LRTI, consulting for the first time for this illness episode EX: immune deficiency	aet prog
Hamre 2005 [35]	Austria, Germany, Netherlands, UK, USA	29 primary care practices with 37 GPs	04/1999–03/2000	prospectively	318 patients (301 after follow-up) ♀ 60%	< 5–65 + 64.9% ≥ 18	IN: age ≥ 1 month, chief complaint of cough ≤ 7 days EX: dementia, renal failure, severe hepatic disease, ongoing immunosuppressive treatment, chemotherapy or radiotherapy, alcohol or drug abuse	prog
Harding 1980 [36]	Colombia, India, Sudan, Philippines	several primary care health facilities	n.r.	prospectively	1624 patients ♀ 75%	n.r.	IN: attending patients ≥ 16 years EX: seriously ill (e.g. coma), requiring urgent medical care	pre
Hofmans-Okkes 1993 Dutch Study [37]	Netherlands	6 practices with 10 physicians	n.r.	prospectively	385 RFE 200 consultations ♀ 62%	Ø 40	IN: doctors coded RFE during encounters	pre
Hofmans-Okkes 1993 International Study [37]	Belgium, Denmark, Israel, Italy, Netherlands, Portugal, Spain, UK, USA	22 physicians	01/1990–02/1991	prospectively	943 RFE 497 consultations ♀ 64%	Ø 38	IN: consecutive routine encounters	pre
Hull 1969 [38]	UK	1 rural general practice with 2 GPs	10/1966–02/1967	prospectively	1000 incidental consultations ♀ 54%	n.r.	IN: consecutive new cases presenting in practice EX: consultations for antenatal, immunization or contraceptive care	pre
Liu 2017 [39]	China	14 community health service centers with 100 GPs in 6 suburban districts of Beijing	12/2014–01/2015	prospectively	10,000 consultations 13,705 RFE ♀ 52,5%	< 35–55 +	IN: consecutive patients’ encounters	pre
Martin 1984 [40]	Saudi Arabia	1 primary care department of a hospital serving a military community in Riyadh	n.r.	prospectively	1000 incidental consultations ♀ 42%	0–45 +	IN: patients presenting for the first time with a problem	pre
Mash 2012 [41]	South Africa	83 primary care clinics, 17 mobile clinics, 12 community health centres; nurse-led with support from doctors	1 year	prospectively	18,856 consultations 31,451 RFE ♀ 66%	< 1–79	IN: all ambulatory patients seen by the health worker	pre
Molony 2016 [42]	Ireland	1 large general practice with 4 GPs in a primary healthcare centre in North Cork	10/2010–10/2014	retrospectively	5100 patients 52,572 consultations 70,489 RFE	0–80 +	IN: doctor-patient face-to-face encounters on all working days and 146 non-working days with documentation of diagnostic code EX: contacts with practice nurse/ practice’s administrative team, telephone or ‘out-of-hours’ contacts	pre
Morrell 1971/1972[43, 44]	UK	1 general practice with 3 GPs	1 year	prospectively	4455 patients 21,098 consultations 5323 new symptoms ♀ 52%	0–65 +	IN: new patient-initiated consultations with symptoms not presented to any doctor in the previous 12 months EX: doctor-initiated consultations	pre aet
Munyati 2005 [45]	Zimbabwe	2 primary health care clinics in Harare	n.r.	prospectively	544 patients ♀ 52% 83% HIV-positive	16–55 + Ø 33	IN: patients ≥ 16 years with cough ≥ 3 weeks consulting on weekdays EX: treatment for tuberculosis; requiring immediate admission to hospital; unwilling to undergo HIV-testing; not resident in region Mbare	aet
NAMCS [46, 47]	USA	general internists, family practioners or general practicioners	1980, 1981, 1985, 1989–1994 resp. 1985–1986	prospectively	3416–183,225 consultations ♀ 59–60%	< 15–75 + resp. 0–75 +	IN: visits by patients with a chief complaint of cough during a randomly assigned 1-week reporting period	pre aet
Nantha 2014 [48]	Malaysia	1 primary health care clinic	01–05/2013	prospectively	151 patients (117 after follow-up) ♀ 49%	18–60 +	IN: patients > 18 years presenting with a chief complaint of cough > 2 weeks	aet
Njalsson 1992 [49]	Iceland	12 rural and 4 urban primary care health centres	01–12/1988	prospectively	49,193 patients 284,348 RFE ♀ 60%	0–75 +	IN: all contacts (including prescriptions, follow-up visits, tests, procedures and administrative visits)	pre
Robertson 1991 [50]	USA	1 GP in 1 Family Medicine Unit at the Medical University of South Carolina	07/1976–06/1979	prospectively	304 patients 956 consultations 1377 RFE	0–65 +	IN: all patient contacts	pre
SESAM 2 Study [51, 52]	Germany	209 GPs in the federal state of Saxony	10/1999–09/2000	prospectively	8877 patients 13,632 RFE ♀56,9%	0–75 +	IN: randomly selected patients presenting in general practice (tenth consultation of the consultation hour) previously known to the practitioner EX: house calls, patients already included in SESAM 2 study	pre aet
Silva 1998 [53]	Sri Lanka	34 general practioners	07/1996	prospectively	2068 consultations 3448 RFE ♀ 53%	< 12–65 +	IN: consecutive doctor-patient encounters	pre
Stefanoff 2014 [54]	Poland	34 health units with 78 GPs	07/2009–04/2011	prospectively	158,863 patients 197,955 py ♀ 52%	3–70 +	IN: patients ≥ 3 years, cough 2–15 weeks	pre aet
TRANSITION Project [37, 55]	Netherlands resp. Netherlands, Malta and Serbia	54 family physicians in 23 locations in the Netherlands resp. family physicians in the Netherlands, Malta and Serbia	1985–1995 resp. 1995–2005	prospectively	93,297–274,620 py 236,027 EOC 267,897–337,348 consultations	n.r.	IN: episode data for all face-to-face encounters with their listed patients	pre aet
Verzantvoort 2018 [56]	Netherlands	users of the smartphone application “Should I see a doctor?” as a self-triage decision tool for acute primary care	07/2014–07/2015	prospectively	4446 app users 3317 patients with registered symptoms ♀ 66%	0–66 +	IN: app-users who answered to have used the app for a current medical problem	pre
Wong 2016 [57]	China	19 clinicians in Hong Kong public primary care clinics and private clinics	11/2011–02/2014	prospectively	455 patients (321 after follow-up) ♀ 57%	Ø47.1	IN: immunocompetent patients ≥ 18 years consulting within normal consulting hours with an acute or worsened cough (≤ 28 days duration) as main symptom, or clinical presentation that suggested LRTI	prog
Woolnough 1985 [58]	Canada	1 family practice	4 separate months in each season of the year	prospectively	32 patients ♀ 59%	20–70 +	IN: all patients whose chief presenting reason was cough	aet
Worrall 2008 [59]	Canada	1 community health centre, 1 GP	fall/winter 2005–2006	prospectively	100 patients	1–90	IN: consecutive patients with cough ≤ 14 days	aet

Legend: aet = aetiology of the symptom cough in primary care, EOC = episode of care, n.r. = not reported, pre = prevalence of the symptom cough in primary care, prog = prognosis of the symptom cough in primaryare, py = patient years, resp. = respectively, RFE = reasons for encounter, ♀ = female, Ø = mean

### Assessment of risk of bias

Depending on the selection of patients and GPs (Domain A) most studies had a low risk of substantial variation and of risk of bias. Referring to data collection and patient flow (Domain B) the risk of bias was found to be low in most studies (*n* = 20), and none had a high risk of bias. In diagnostic workup (Domain C) most showed a high risk of bias (*n* = 7). The risk of bias in the prognostic workup (Domain D) was low in one study, unclear in another, and had different assessments in two studies, depending on the prognostic category. Only seven studies had an overall low risk of bias. A summary is presented in Table 3; detailed methodological description and risk of bias can be found in Additional File 2.

**Table 3:** Assessment of substantial variation and risk of bias

Domain Study	A: Substantial variation in selection of patients and GPs^1^	A: Risk of bias in selection of patients and GPs^1^	B: Risk of bias in data collection and patient flow^1^	C: Risk of bias in diagnostic work-up^2^	D: Risk of bias in prognostic work-up^3^
Ajmi 2011 [16]	low	?	low	n.r.	n.r.
Albert 2011 [17]	high	high	?	n.r.	n.r.
BEACH	low	low	low	n.r.	n.r.
Ben Abdelaziz 2004 [19]	low	?	low	n.r.	n.r.
Coenen 2004 [20]	?	low	?	n.r.	?
CONTENT	low/?*	?	?	high	n.r.
French 2005 [23]	low	high	low	?	n.r.
GRACE	?/high*	low	low/?*	low/?/high*	?/high*
Hamre 2005 [35]	?	low	low	n.r.	low
Harding 1980 [36]	?	low	low	n.r.	n.r.
Hofmans-Okkes 1993 International Study	?	low	low	n.r.	n.r.
Hofmans-Okkes 1993 Dutch Study	?	?	low	n.r.	n.r.
Hull 1969 [38]	?	high	low	n.r.	n.r.
Liu 2017 [39]	low	high	low	n.r.	n.r.
Martin 1984 [40]	high	high	low	n.r.	n.r.
Mash 2012 [41]	high	low	low	n.r.	n.r.
Molony 2016 [42]	low	high	?	n.r.	n.r.
Morrell 1971/1972 [43, 44]	high	high	low	high	n.r.
Munyati 2005 [45]	high	high	low	low	n.r.
NAMCS	low	low/?*	low/?*	high	n.r.
Nantha 2014 [48]	low	high	?	?	n.r.
Njalsson 1992 [49]	low	low	?	n.r.	n.r.
Robertson 1981 [50]	low	high	low	n.r.	n.r.
SESAM 2	low/high*	low	low	high	n.r.
Silva 1998 [53]	low	low	low	n.r.	n.r.
Stefanoff 2014 [54]	?	?	?	?	n.r.
TRANSITION	low	low	low	high	n.r.
Verzantcoort 2018 [56]	high	low	low	n.r.	n.r.
Wong 2016 [57]	?	low	?	n.r.	low/?*
Woolnough 1985 [58]	?	high	?	low	n.r.
Worrall 2008 [59]	low	high	low	high	n.r.

Legend: ? = unclear, n.r. = not relevant, 1 = refers to all included studies, 2 = refers solely to studies that present data on the underlying aetiologies of cough patients, 3 = refers solely to studies that present prognostic outcomes, * = varying assessments for different publications or different aetiological /prognostic categories

### Prevalence and incidence

**Fig. 2:**
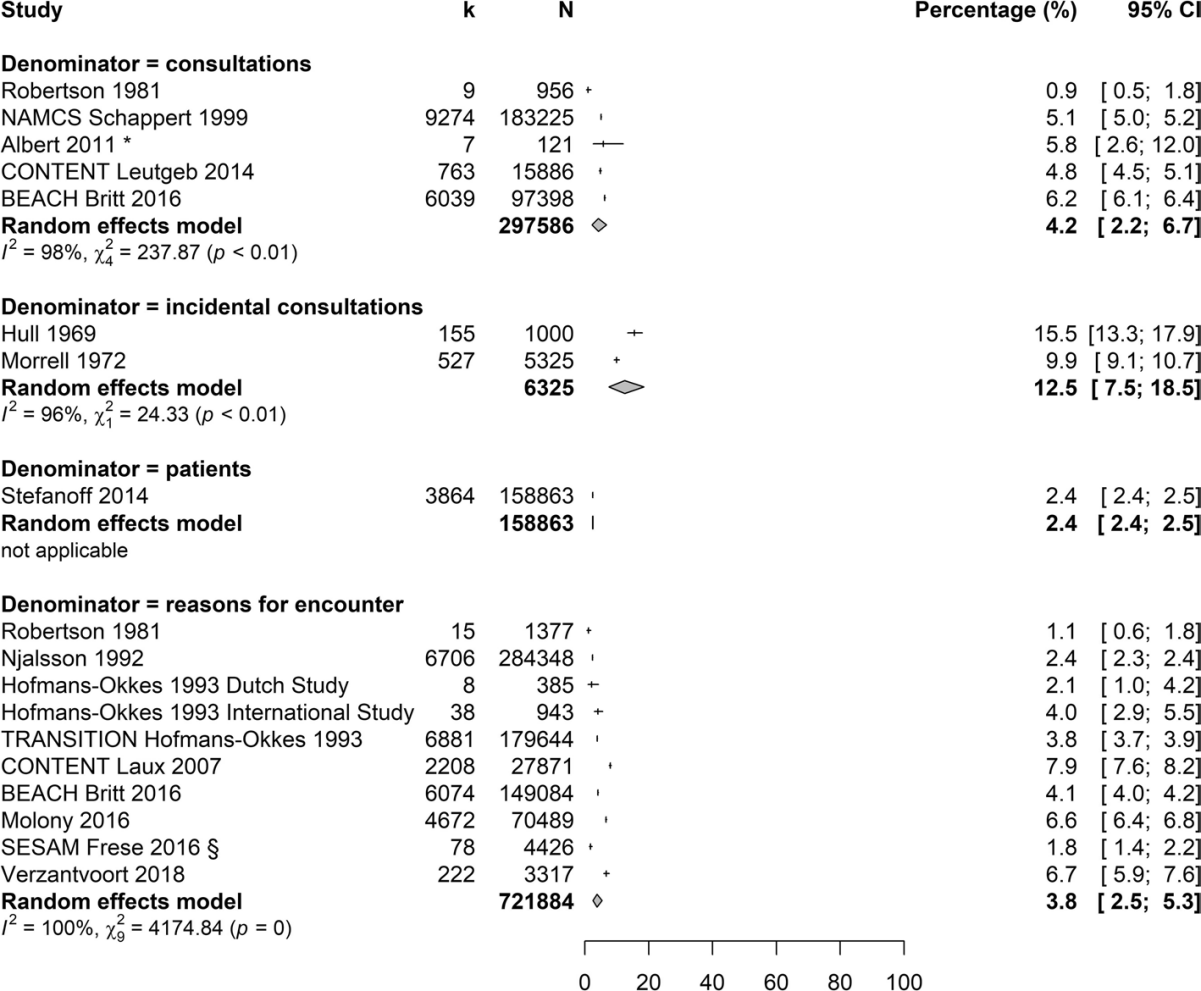
Meta-analysis: prevalence/incidence of cough of all durations in Western countries sorted by denominators. Estimates refer to consulting primary care patients of all age groups. * = study included adults only, § = study included patients ≥ 65 years, CI = confidence interval, k = number of (incidental) consultations because of cough / patients in consultation for cough / reason for encounter = cough, N = total number of (incidental) consultations / patients in consultation / reasons for encounter

Twenty-two studies presented outcomes on the prevalence of cough; nine of these show a low risk of bias. Figure 2 presents the prevalences and incidences of cough in Western primary care. Incidental consultations showed about three times as many estimates in comparison with prevalences. Outliers were characterized by study populations recruited in a single primary care practice with one or two GPs [38, 50] or by excluding consultations for cough of < 2 and > 15 weeks duration [54]. Comparably low prevalences were seen in a study population of patients aged ≥ 65 years [51] and in studies including not only consultations for symptoms, but also for prescriptions, follow-up visits, tests, procedures and administrative visits to the denominator [49, 50].

Studies with data collection in African, Asian and South American primary care settings show higher estimates of prevalence (13.8% for reasons for encounter and 10.3% for patients), while they show lower estimates of incidence (6.3% for consultations) (see Additional File 3). The presented estimates show a high heterogeneity across studies, indicated by high values of I^2^ and χ^2^.

### Aetiology

**Fig. 3:**
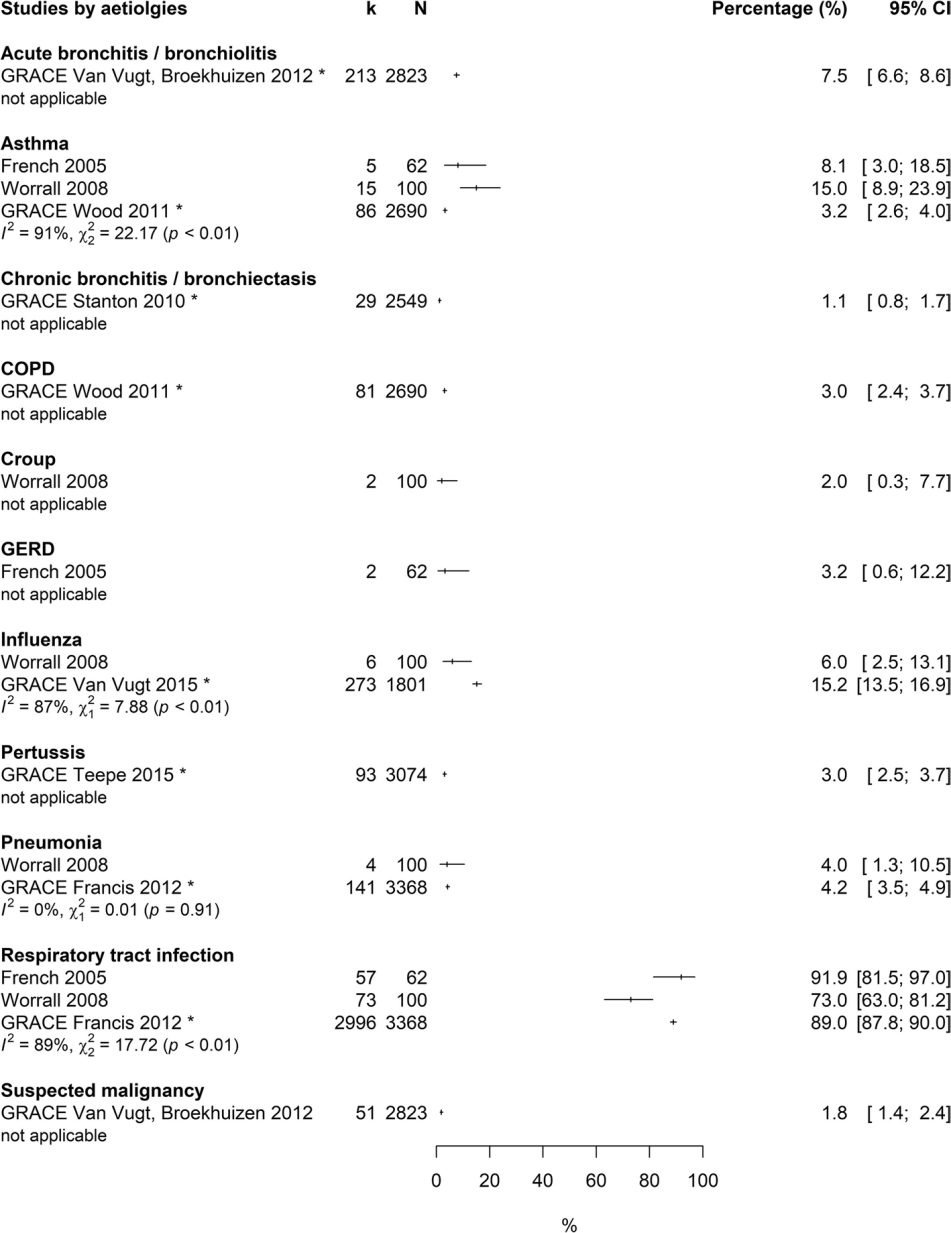
Forest plot: Prevalences of selected aetiologies in patients with acute cough. Estimates refer to primary care patients of all age groups in consultation for acute cough. Denominator: patients. * = GRACE Study included adults only, CI = confidence interval, GERD = Gastroesophageal reflux disease, k = number of patients with the respective aetiology, N = total number of patients in consultation for cough

**Fig. 4:**
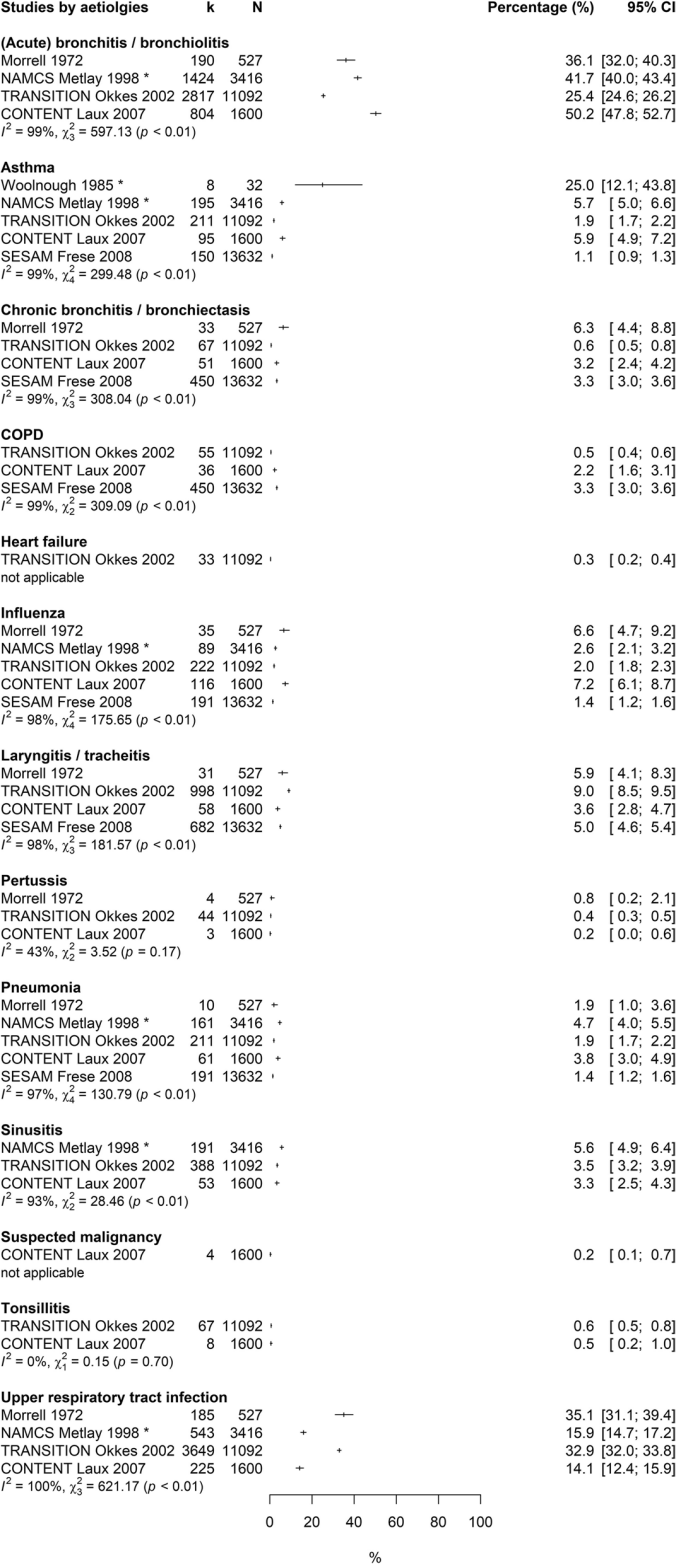
Forest plot: Prevalences of selected aetiologies in patients with cough of all durations. Estimates refer to primary care patients of all age groups in consultation for cough of all durations. Denominators: Consultations (NAMCS Metlay 1998), episodes of care (TRANSITION Okkes 2002), incidental consultations (Morrell 1972), reasons for encounter (CONTENT Laux 2007, SESAM Frese 2008), patients (Woolnough 1985). * = studies included adults only, CI = confidence interval, COPD = Chronic obstructive pulmonary disease, k = number of patients with the respective aetiology, N = total number of patients in consultation for cough

Twelve studies assessed data on the aetiology of cough in primary care. Data referred to different durations of cough and a wide spectrum of differential diagnoses. Mostly, the given aetiologies were the working or presumptive diagnoses by the treating GPs, which correlate with a high risk of bias in the diagnostic workup process. No study had a low risk of bias in all categories. As there were differing denominators (reasons for encounter, (incidental) consultations, episodes of care, patients), no meta-analysis was performed and data is presented in forest plots (Fig. 3, Fig. 4). Data on acute cough and cough of all durations were collected in North America and Europe. The most frequent underlying conditions in acute cough were respiratory tract infections (ranging from 73–91.9%) and in cough of all durations, bronchitis/bronchiolitis (25.4–50.2%). Potentially serious diseases like pneumonia, chronic obstructive pulmonary disease (COPD), heart failure or suspected malignancy were rare. Findings on subacute/chronic cough derived from a study conducted in Zimbabwe (with an HIV prevalence of 83%) [45] and Malaysia [48], showing high prevalences of tuberculosis (6.0–43.0%) and pneumonia (2.8–16.0%) (see Additional File 4). The results of these studies are not applicable to the context of Western countries. The high quality study by Munyati et al. [45] is based on a sample with 83% HIV positive patients; the work by Nantha et al. [48] lacks sufficient information to estimate the risk of bias. In the foremost aetiological categories, we found substantial heterogeneity across studies, indicated by high values of I^2^ and χ^2^.

### Prognosis

Four studies assessed prognostic outcomes, one with an overall low risk of bias. Studies included patients with acute cough of up to one [35] or four weeks [20, 30–34, 57]. The follow-up duration was 28 days in all studies, assessed by a symptom diary or telephone interview.

The median duration of cough after first consultation was reported to be eight (IQR 6–14.5) days [30], with the median time to feeling recovered 9 [57] to 11 days. [34] The mean total illness duration was 20.4 days (standard deviation 10) in patients who felt recovered after four weeks [31]. A first improvement of cough was seen the third day after consultation in 52% of patients [35]. A major improvement or complete recovery was seen in 65.7% of patients after seven days and in 81.4% after 14 days [35]. 10.8% of patients felt completely recovered after seven days [35], 40.2% [35] to 67% [32] of patients after 14 days, and 79% [31] after 4 weeks. A prolonged illness (moderate or severe symptoms more than 3 weeks after consultation), was described in 7.9% of patients [32]. At day 28 after the first consultation, 21.3% of patients still didn’t feel recovered [31]. The re-consultation rate ranged from 21.1% [20] to 35% [30, 32]. Most patients re-consulted the GP during working hours (27.6%), 1.4% out of hours, 2.8% consulted a nurse, 2.7% a specialist, 0.5% a hospital emergency department and 17.2% visited a pharmacist [30]. Between 0% [30] and 1.3% [57] of patients were hospitalized for 3–3.5 days [57] because of cough. No patient died of cough during follow-up [32, 33].

## Discussion

### Main findings

Our study identified 31 studies evaluating the symptom cough in primary care. Data quality was heterogeneous with only seven studies having an overall low risk of bias. The prevalence of cough in Western primary care was 3.8–4.2%; the incidence was 12.5%. African, Asian and South American healthcare settings showed higher prevalences (10.3–13.8%) and lower incidences (6.3–6.5%). Respiratory tract infection (73–91.9%) was the most frequent aetiology in patients with acute cough; bronchitis/bronchiolitis was the most frequent aetiology (25.4–50.2%) in patients with cough of any duration. Other frequent underlying conditions in both were influenza (6–15.2%), asthma (3.2–15.0%), and laryngitis/tracheitis (3.6–9.0%). Serious diseases like pneumonia (4.0–4.2%), COPD (0.5–3.3%), heart failure (0.3%) and suspected malignancy (0.2–1.8%) were rare. Findings on subacute or chronic cough were based on two studies conducted in Zimbabwe and in Malaysia, showing high prevalences of infectious diseases (tuberculosis and pneumonia). For acute cough patients, the median time to feel recovered was 9 to 11 days. Complete recovery was reported by 40.2- 67% of patients after two weeks (79% after four weeks). 21.1- 35% of patients re-consulted, 0–1.3% were hospitalized and none died.

### Prevalence

To our knowledge, there are no other reviews estimating the prevalence or incidence of cough in primary care. However, evidence is needed to set focus in priorities for research, resources, policy making, guideline development and training of primary care professionals [60]. In comparison with our data, the prevalence of cough in population-based surveys is higher (9% to 33%) than in primary care [1], most likely due to its self-limiting course. A population-based telephone survey in Italy showed that 23% of subjects would use domestic remedies, 21% would ask their pharmacist and only 33% would consult their doctor [61]. However, when it comes to consultation, for the majority of people (69.6%-73.7%) the GP is the first address [61, 62].

In Western countries, differences between prevalence and incidence estimates were quite high, with prevalences of about 4% and incidence at 12.5%. This is different in African, Asian and South American primary care settings (10.3–13.8% prevalence and 6.3–6.5% incidence). This might possibly be attributed to the high share of chronic diseases in Western countries, in relation to which cough is less relevant than when compared to a population with a high share of acute diseases. Moreover, study outcomes depend on cultural variance between countries (e.g. different healthcare systems, the patient’s own health traditions, and different thresholds for consulting a doctor) [14]. In developing countries with a higher rate of uninsured people and fewer health care providers (especially in rural areas) there are fewer consultations for self-limiting acute respiratory tract infections. Furthermore, environmental factors associated with poverty (cooking on an open fire and a higher burden of HIV-infections, accompanied by higher rates of tuberculosis) increase the prevalence of chronic cough.

### Aetiology

International guidelines suggest classifying cough according to its duration, as either acute (< 3 weeks), subacute (3–8 weeks), or chronic cough (> 8 weeks) [5, 6, 63, 64], or as acute and chronic cough [7, 65–67]. In fact, the most common definition for chronic cough is ≥ 3 months duration [68]. A categorisation seems necessary as acute cough is mostly caused by a respiratory tract infection, usually vanishing within two weeks [1]. In contrast, chronic cough is associated with a greater risk of serious diseases that require efficient treatment or referral [6]. This is confirmed by our results: we found respiratory tract infections to be the most common underlying conditions of acute cough, followed by exacerbations of asthma and influenza. This is in accordance with primary care guidelines recommending that laboratory tests, sputum evaluation, chest x-rays, and antibiotic treatment all be foregone when respiratory tract infection is clinically likely and no warning signs of serious disease are present [69].

Our results concerning aetiologies of chronic cough are based mainly on two studies from Malaysia [48] and Zimbabwe [45], with a cough > 2/ ≥ 3 weeks. Other than a study from Poland, assessing the prevalence of pertussis [54], we didn’t find any evidence for chronic cough in Western primary care and none concerning subacute cough. Our data do not confirm the big three causes of chronic cough (Chronic upper airway cough syndrome, asthma, and gastroesophageal reflux disease (GERD), nor any other differential diagnosis. The respective recommendations on subacute or chronic cough are based on secondary or tertiary care studies [6, 70]. In fact, given the different case mix, it is likely that the distribution of causes is different in primary care.

### Prognosis

Accurate prediction of the course of cough could decrease antibiotic overprescribing [71, 72]. Half of antibiotic prescriptions for acute respiratory conditions in US ambulatory care visits seem to be unnecessary [73]. About 53% of acute cough patients in Europe receive antibiotics [34] – despite the high prevalence of underlying self-limiting viral infection [6, 74]. We found no death, a low rate of hospital admissions, an improvement in half of patients after three days and complete recovery in 79% of patients after one month. A benign course of acute cough was also found by Bruyndonckx et al. [71]. A systematic review assessing primary, secondary, and tertiary care found a weighted mean duration of any cough of 17.8 days (range 15.3 to 28.6 days) and 13.9 days for productive cough (range 13.3 to 17.4 days) [75]. In our study the mean total illness duration was 20.4 days (standard deviation 10). As for acute cough, symptom control without diagnosis ('wait and see approach') seems more sensible than investing in unnecessary diagnostic resources [76]. To reassure patients with low risk, and to confine patients with a high risk of complication, primary care prediction tools like RISSC85 [71] are helpful.

We didn’t identify any studies presenting evidence on prognostic outcomes concerning subacute or chronic cough in primary care; this should be addressed in future research.

Guidelines define cough of more than eight weeks as chronic [6, 63, 64]. In fact, the longest follow-up in prognostic studies was 28 days. Outcome assessment varied vastly across prognostic studies; accordingly, standardization seems mandatory. None of the included prognostic studies contained an untreated or alternative control group, leading to a high risk of bias.

### Strength and limitations of our study

Our work comes at a time when the epidemiology of cough has shifted due to the Covid 19 pandemic. Struyf et al. [77] performed a systematic review over the accuracy of Covid-19 symptoms in primary care and in hospital outpatient settings. They identified 44 studies, including three from primary care settings. In a sample including 21% patients suffering from Covid19, they found 65% of patients presenting with cough, of whom 142 would have Covid-19. The search strategy (searching for Covid-19 studies) was different from our study design and symptoms were actively asked for, so frequencies are overestimated. But even if the study had fit our requirements, these data would be outliers. During a pandemic, the prevalence of diseases and symptoms shifts. In addition, the utilization behaviour, the diagnostics and the frequency of aetiologies as well as the morbidity change. Interventions related to Covid-19 like facial masks are displacing diseases such as influenza and, at the same time, pneumonia is increasing as a cause of cough due to viral illness. Studies conducted during the pandemic are not comparable to the everyday situation of a family practice, which we would like to depict in our review. We must point out that the results of our study apply only to the periods leading up to the pandemic. After that, it will be important to examine whether behavioural changes (such as refraining from shaking hands) as a result of the pandemic will change the observed epidemiological data in our study.

Apart from this temporal classification, we must consider the typical weaknesses of a systematic review. Conclusions of any systematic review can only be as valid as the available literature and the accuracy of the included studies’ protocols [75]. Important aspects are (1) limitations to the internal validity of the included studies (e.g. imprecise inclusion criteria or incomplete recruitment); (2) criteria affecting the external validity of the included studies (e.g. characteristics of the setting, or recruitment practice); (3) methodological aspects of our review affecting the internal validity of our review (e.g. accuracy in literature search, screening process or data analysis); (4) aspects influencing the review’s external validity [10, 13].

Accordingly, we performed strict quality assessment and implemented clear inclusion criteria. Our research was comprehensive and thorough, with almost all abstracts and full texts screened by two reviewers. To minimize selection bias, we excluded all studies that explicitly included or excluded certain groups of cough patients and we contacted study authors to acquire missing information. Still, in some cases uncertainty remained regarding eligibility criteria, definition of outcomes or denominators of given data. This may have introduced error into our data synthesis.

We didn’t control the risk of bias across studies and the publication bias, as the number of studies concerning the respective outcome was too low. However, it is rather unlikely that prevalences of cough or underlying conditions are not published.

Limitations to our review are the substantial methodological and clinical heterogeneity across included studies. As Higgins et al. postulated “every amount of heterogeneity is acceptable, providing both that the predefined eligibility criteria for the meta-analysis are sound and that the data are correct” [78]. We built subgroups referring to denominators, duration of cough and cultural variances in healthcare systems. In aetiological outcomes, the formation of categories was difficult and overlapping of categories is likely. Given (sub-)categories differed widely. Denominators weren’t always specified, which may have influenced data synthesis.

The attribution of countries to the subgroups *Western* resp. *African/Asian/South American countries* corresponds with the United Nations classification system of *developed* and *developing countries* [79]. We didn’t use the latter terms, because people’s health demands depend not only on the economic situation of a country, but also on health systems, people’s health convictions and utilization of health care.

The assessment of the methodological quality and the risk of bias should be based on standardized checklists. Yet, there are no published criteria referring to studies evaluating symptoms [13]. Therefore our research group has developed a tool for assessing methodological quality and risk of bias, based on work done by Donner-Banzhoff et al. and on the Standards for the Reporting of Diagnostic Accuracy (STARD) on diagnostic accuracy studies [8, 80]. Applying our tool, we found an overall low risk of bias in only ten studies with prevalence outcomes and in one study with prognostic outcomes, while there was no such study presenting aetiological results. The latter is caused mainly by the fact that the majority of aetiological studies evaluated clinical diagnoses without a standardised diagnostic approach or follow-up. Despite these limitations, most studies in subgroups had similar results, and we think our results are currently the best approach wehave to guide the GP in his everyday decisions.

Statistical limitations can be quantified. Content-related aspects can only be discussed and made transparent. We discussed seasonal effects and differences between countries. We ourselves see no reason to exclude older studies as long as they meet the inclusion criteria, and as long as their sample shows an appropriate external validity. This would be different if we knew of any event that calls into question the epidemiological situation at the time, but as far as we know there is nothing we have to consider. If we were already 10 years further along, we would probably exclude the studies of today because of the special situation under pandemic conditions.

## Conclusions

In conclusion, we found cough to be a common reason for consulting in primary care. In the majority of patients presenting for an acute cough, underlying conditions are respiratory tract infections with a benign self-limiting course. About 80% of these patients show an improvement of symptoms within three days and a complete recovery after 4 weeks, which supports a wait-and-see approach at an early stage of disease. Studies on asthma or influenza show substantial variation of frequencies (3–15%, resp. 6–15%). Potentially serious diseases like malignancy or pneumonia occur with less than 1% (resp. 4%) in acute cough. In General Practice the duration of cough is a strong diagnostic tool to distinguish between benign courses and diseases that are more serious. However, since there is no subgroup specific aetiological evidence for prolonged or chronic cough, we cannot capture the changes in pre-test probabilities over time in our data, which is mandatory for GPs’ diagnostic workup. For future studies, we see a particular need in methodologically sound studies on the cause of subacute and chronic cough in Western primary care. Family physicians need this data to carry out their filtering and pick-up function in the healthcare system. Our study reflects the realities of primary care under non-pandemic conditions. It will be interesting to examine the epidemiological impact of the pandemic on the new normal and compare it with our results.

## Supplementary Information

**Additional file 1.** Detailed search strategy.

**Additional file 2.** Assessment of methodological quality, risk of bias and sources of clinical heterogeneity.

**Additional file 3.** Meta-analysis: Prevalence / incidence of cough in African, Asian and South American countries.

**Additional file 4.** Aetiologies of subacute and chronic cough.

## Data Availability

All data analysed during this study were drawn from published articles. The respective references and extracted numbers are all included in this article and its additional information files.

## Abbreviations

ACE: Angiotensin-Converting-Enzyme
aet: Aetiology
CI: Confidence Interval
COPD: Chronic Obstructive Pulmonary Disease
EOC: Episode Of Care
GERD: Gastroesophageal Reflux Disease
GP: General Practitioner
n.r.: Not reported
pre: Prevalence
prog: Prognosis
py: Patient years
resp.: Respectively
RFE: Reason For Encounter
♀: Female
Ø: Mean
