# Supplementary material for: Prevalence, aetiologies and prognosis of the symptom cough in primary care: a systematic review and meta-analysis
Source: BMC Fam Pract. 2021 Jul 12;22:151. doi: 10.1186/s12875-021-01501-0 (PMC8274469; doi:10.1186/s12875-021-01501-0)
Supplement: Supplementary file 2 — Additional file 2. Assessment of methodological quality, risk of bias and sources of clinical heterogeneity. [file 12875_2021_1501_MOESM2_ESM.pdf]

Additional file 2: Assessment of methodical quality, risk of bias and sources of clinical heterogeneity

Instrument:

For each domain, reviewers independently answered the standardized key questions (yes, no or unclear) and assessed the risk of bias for the respective domain (low, high or unclear). In Domain A reviewers also rated their concern that the selection of patients and/or of GPs may have introduced substantial variation/ clinical heterogeneity (low, high or unclear).
Domain A and B referred to all studies regardless of the respective study outcome. Domain C was only considered if the respective study reported data on the underlying aetiologies of cough patients. Domain D was only considered if the respective study presented prognostic outcomes. In Domain C and D, key questions had to be answered separately for each diagnostic or prognostic category respectively.

<table>
<tr><td colspan="2">Domain A: Selection of patients and GPs (refers to all studies regardless of the review question)</td></tr>
<tr><td>Item 1</td><td>Was the symptom to be investigated clearly described?</td></tr>
<tr><td>Item 2</td><td>Were the selection criteria of the patients clearly described?</td></tr>
<tr><td>Item 3</td><td>Was a consecutive or random sample of patients enrolled?</td></tr>
<tr><td>Item 4</td><td>Was it a multi-centre study?</td></tr>
<tr><td colspan="2">Judgement: Risk that the selection of patients introduced bias: low, unclear, high</td></tr>
<tr><td>Item 5</td><td>Did the selection criteria of the patients permit the study population to represent the full spectrum of those presenting with the symptom in the respective setting/ addressed in the review question?</td></tr>
<tr><td>Item 6</td><td>Were the participating health care professionals/ institutions representative for the setting to be investigated in the review.</td></tr>
<tr><td colspan="2">Judgement: Concern that the selection of patients and GPs introduced substantial variation or clinical heterogeneity: low, unclear, high</td></tr>
<tr><td colspan="2">Domain B: Data collection and patient flow (refers to all studies regardless of the review question)</td></tr>
<tr><td>Item 7</td><td>Were data about the symptom und the inclusion criteria collected directly from the patients (as opposed to a proxy like a register, routine documentation)</td></tr>
<tr><td>Item 8</td><td>Was the same mode of data collection used for all patients?</td></tr>
<tr><td>Item 9</td><td>Was the number of non-responders/ dropouts unlikely to affect the results?</td></tr>
<tr><td colspan="2">Judgement: Risk that the mode of data collection and/ or patient flow introduced bias: low, unclear, high</td></tr>
<tr><td colspan="2">Domain C: Determination of the underlying aetiology/ diagnostic work-up (refers only to review question “What are the underlying conditions and their respective frequencies (differential diagnosis)?”). Had to be answered for each diagnostic category separately.</td></tr>
<tr><td>Item 10</td><td>Was the aetiologic category clearly defined?</td></tr>
<tr><td>Item 11</td><td>Was the diagnostic work up likely to correctly classify the respective aetiology?</td></tr>
<tr><td>Item 12</td><td>Did every patient receive the same diagnostic work up to detect the respective aetiology?</td></tr>
<tr><td colspan="2">Judgement: Risk that the diagnostic work up introduced bias: low, unclear, high</td></tr>
<tr><td colspan="2">Domain D: Determination of the prognosis/ prognostic work-up (refers only to review question “What is the prognosis of patients with the respective symptom presenting in the respective setting?”)
Had to be answered for each prognostic category separately.</td></tr>
<tr><td>Item 13</td><td>Was the prognostic outcome clearly defined?</td></tr>
<tr><td>Item 14</td><td>Did the study design include a comparison group without the symptom?</td></tr>
<tr><td>Item 15</td><td>Was the work up/ measurement likely to correctly classify the respective prognostic outcome?</td></tr>
<tr><td>Item 16</td><td>Did every patient receive the same work up/ mode of data collection to verify the respective prognostic outcome?</td></tr>
<tr><td colspan="2">Judgement: Risk that the prognostic work up introduced bias: low, unclear, high</td></tr>
</table>

Detailed results:

Domain A (Selection of patients and GPs) and domain B (Data collection and patient flow)

	**Domain A**								**Domain B**			
**Study**	Item 1	Item 2	Item 3	Item 4	Risk of bias in selection of patients and GPs	Item 5	Item 6	Substantial variation in selection of patients and GPs	Item 7	Item 8	Item 9	Risk of bias in data collection and patient flow
Ajmi 2011	-	-	?	+	?	+	+	low	?	+	+	low
Albert 2011	-	-	+	-	high	-	-	high	+	+	-	?
BEACH	-	?	+	+	low	+	+	low	+	+	+	low
Ben Abdelaziz 2004	-	+	+	-	?	+	+	low	+	+	+	low
Coenen 2004	+	+	+	+	low	-	+	?	+	+	-	?
CONTENT Laux 2007	-	-	?	+	?	+	+	low	-	+	+	?
CONTENT Leutgeb 2014	-	?	?	-	?	+	-	?	-	+	+	?
French 2005	+	+	+	-	high	+	+	low	+	+	+	low
GRACE<br>Butler 2009;<br>Godycki-Cwirko 2011;<br>Stanton 2010;<br>Van Vugt, Butler 2012	+	+	+	+	low	?	+	?	+	+	-	?
GRACE<br>Francis 2012;<br>Hordijk 2014;<br>Van Vugt 2015;<br>Wood 2011	+	+	+	+	low	?	+	?	+	+	+	low
GRACE Teepe 2015	+	+	+	+	low	?	+	?	+	+	?	low
GRACE<br>Van Vugt, Broekhuizen 2012;<br>Van Vugt, Broekhuizen 2013	+	+	+	+	low	-	+	high	+	+	+	low
Hamre 2005	+	+	+	+	low	?	?	?	+	+	+	low
Harding 1980	-	+	+	+	low	+	?	?	+	+	+	low
Hofmans-Okkes 1993<br>International Study	-	-	+	+	low	?	?	?	+	+	+	low
Hofmans-Okkes 1993<br>Dutch Study	-	-	?	?	?	?	?	?	+	+	+	low
Hull 1969	+	+	+	-	high	?	+	?	+	+	+	low
Liu 2017	-	?	+	-	high	+	+	low	+	+	+	low
Martin 1984	-	+	?	-	high	?	-	high	+	+	+	low
Mash 2012	-	?	+	+	low	+	-	high	+	+	+	low
Molony 2016	-	+	+	-	high	+	+	low	-	+	+	?
Morrell 1971/1972	-	+	?	-	high	-	?	high	+	+	?	low
Munyati 2005	+	+	+	-	high	-	+	high	+	+	+	low
NAMCS Metlay 1998	-	-	?	+	?	+	+	low	+	+	?	?
NAMCS Schappert 1999	-	?	+	+	low	+	+	low	+	+	+	low
Nantha 2014	+	-	?	-	high	+	+	low	+	+	-	?
Njalsson 1992	-	-	?	+	low	+	+	low	-	+	+	?
Robertson 1981	-	-	+	-	high	+	+	low	+	+	+	low
SESAM 2 Frese 2008	-	+	+	+	low	+	+	low	+	+	+	low
SESAM 2 Frese 2016	-	+	+	+	low	-	+	high	+	+	+	low
Silva 1998	-	+	+	+	low	+	+	low	+	+	+	low
Stefanoff 2014	+	?	?	+	?	?	?	?	+	+	-	?
TRANSITION<br>Hofmans-Okkes 1993	-	-	+	+	low	+	+	low	+	+	+	low
TRANSITION Okkes 2002	-	?	+	+	low	+	+	low	+	+	?	low
Verzantvoort 2018	-	?	+	+	low	?	-	high	+	+	+	low
Wong 2016	+	+	+	+	low	?	+	?	+	+	-	?
Woolnough 1985	-	-	+	-	high	?	+	?	+	+	?	?
Worrall 2008	+	-	+	-	high	+	+	low	+	+	+	low

Legend: + Yes, - No, ? Unclear

Domain C (Determination of the underlying aetiology of cough / diagnostic work-up)

		Domain C			
**Study**	**Aetiologic category**	Item 10	Item 11	Item 12	Risk of bias in diagnostic work-up
CONTENT Laux 2007	R78 acute bronchitis / bronchiolitis, R96 asthma, R79 chronic bronchitis, R95 COPD, R80 influenza, R77 laryngitis / tracheitis acute, R71 pertussis, R81 pneumonia, R75 sinusitis acute / chronic, R84 malignant neoplasm bronchus/lung, R76 tonsillitis acute, R74 Upper respiratory infection acute (ICPC process codes)	-	?	-	high
French 2005	asthma, GERD, URTI	-	?	?	?
GRACE Francis 2012	LRTI, URTI, viral / other RTI, pneumonia	-	?	-	high
GRACE Stanton 2010	chronic bronchitis / bronchiectasis	-	?	-	high
GRACE Teepe 2015	pertussis	-	+	+	low
GRACE Van Vugt, Broekhuizen 2012	acute bronchitis / bronchiolitis	-	-	+	high
	suspected malignancy	+	?	+	?
GRACE Van Vugt 2015	influenza	-	+	+	low
GRACE Wood 2011	asthma, COPD	-	?	-	high
Morrell 1972	acute bronchitis, chronic bronchitis, common cold, influenza, laryngitis / tracheitis, pertussis, pneumonia	-	?	-	high
Munyati 2005	asthma, cryptococcosis, fibrotic lung disease (post-tuberculous disease, idiopathic diffuse fibrosis), heart failure, HIV-associated, LRTI, pneumocystis pneumonia, bacterial pneumonia, tuberculosis, cancer (pulmonary / cutaneous Kaposi sarcoma)	+	+	+	low
NAMCS Metlay 1998	acute bronchits / bronchitis not specified as acute or chronic 466/490, URTI 465, asthma 493, acute / chronic rhinosinusitis 461/473, pneumonia 481-483/485-486, influenza 487, pharyngitis 462, nasopharyngitis / common cold 460 (ICD-9-CM process codes)	-	?	-	high
Nantha 2014	ACE-inhibitor induced, bronchial asthma, acute bronchitis, COPD, GERD, heart failure, pneumonia, post infectious cough, lung cancer, pulmonary tuberculosis, upper airway cough syndrome	+	+	?	?
SESAM 2 Frese 2008	acute laryngitis / tracheitis, exacerbation chronic bronchitis / COPD, influenza, pneumonia, asthma	-	?	-	high
Stefanoff 2014	pertussis	+	+	-	high
TRANSITION Okkes 2002	R74 upper respiratory infection (head cold), R78 acute bronchitis / bronchiolitis, R77 acute laryngitis / tracheitis, R75 sinusitis acute / chronic, R80 influenza (proven), R96 asthma, R81 pneumonia, R76 tonsillitis acute, R91 chronic bronchitis / bronchiectasis, R95 emphysema /COPD, R71 whooping cough, K77 heart failure (ICPC process codes)	-	?	-	high
Woolnough 1985	bronchospasm	+	+	+	low
Worrall 2008	asthma, croup, influenza, pneumonia, viral RTI	-	?	-	high

Legend: + Yes, - No, ? Unclear, ACE = Angiotensin-converting-enzyme, COPD = chronic obstructive pulmonary disease, GERD = gastroesophageal reflux disease, ICD-9-CM = The International Classification of Diseases 9th revision Clinical Modification, ICPC = International Classification of Primary Care, LRTI = lower respiratory tract infection, RTI = respiratory tract infection, URTI = upper respiratory tract infection

Domain D (Determination of the prognosis / prognostic work-up)

		Domaine D				
**Study**	**Prognostic category**	Item 13	Item 14	Item 15	Item 16	Risk of bias in prognostic work-up
Coenen 2004	reconsultation, hospitalization	+	-	?	?	?
GRACE Butler 2009	median time to patients reporting feeling recovered, median time for patients' symptom severity scores to drop to 0	+	-	+	+	low
	admitted to hospital	+	-	?	+	?
GRACE Coenen 2013	median time for patients' symptom severity scores to drop to 0	+	-	+	+	low
GRACE Godycki-Cwirko 2011	reported recovery, median duration of cough after presentation	+	-	+	+	low
	admitted to hospital, reconsultation	+	-	?	?	?
GRACE Hordijk 2014	hospitalized for this illness episode during the 28 day follow up period, reconsulted their GP for this LRTI episode, absent of work/school for a mean of	+	-	?	?	?
	mean symptom severity score at day 1, mean symptom severity score at day 28, streepest decline in symptom scores, felt recovered after 4 weeks, not feeling recovered at 28 days, total illness duration,mean	+	-	+	+	low
GRACE Van Vugt, Butler 2012	hospitalized, died, re-consultation rate	+	-	?	?	?
	stated they felt recovered at 14 days, prolonged illness (>3 weeks)	+	-	+	+	low
GRACE Van Vugt, Broekhuizen 2013	mortality, admitted to hospital	+	-	?	?	?
Hamre 2005	at day 1: first improvement, at day 3: first improvement, at day 7: major improvement + complete recovery, at day 7: complete recovery, at day 14: major improvement + complete recovery, at day 14: complete recovery	+	-	+	+	low
Harnden 2006	total duration of cough, 2 months after the start of their symptoms still coughing	+	-	+	+	low
Wong 2016	mean time for cough recovery	+	-	+	+	low
	hospitalisation since first consult, average days of hospitalisation, re-consultation to health professional	+	-	?	?	?
Legend: + Yes, - No, ? Unclear						
