# Supplementary material for: Prevalence, aetiologies and prognosis of the symptom cough in primary care: a systematic review and meta-analysis
Source: BMC Fam Pract. 2021 Jul 12;22:151. doi: 10.1186/s12875-021-01501-0 (PMC8274469; doi:10.1186/s12875-021-01501-0)
Supplement: Supplementary file 3 — Additional file 3. Meta-analysis: Prevalence / incidence of cough in African, Asian and South American countries. [file 12875_2021_1501_MOESM3_ESM.pdf]

Additional file 3: Meta-analysis: Prevalence/incidence of cough in African, Asian and South American countries

Study	k	N	Percentage (%)	95% CI
**Denominator = incidental consultations**				
Martin 1984	65	1000	6.5	[5.1; 8.3]
**Random effects model**		**1000**	**6.5**	**[5.1; 8.1]**
not applicable				
**Denominator = incidental reasons for encounter**				
Martin 1984	63	1000	6.3	[4.9; 8.0]
**Random effects model**		**1000**	**6.3**	**[4.9; 7.9]**
not applicable				
**Denominator = patients**				
Harding 1980	167	1624	10.3	[8.9; 11.9]
**Random effects model**		**1624**	**10.3**	**[8.9; 11.8]**
not applicable				
**Denominator = reasons for encounter**				
Silva 1998	576	3448	16.7	[15.5; 18.0]
Ben Abdelaziz 2004	892	6576	13.6	[12.8; 14.4]
Ajmi 2011	4228	24882	17.0	[16.5; 17.5]
Mash 2012	2821	31451	9.0	[8.7; 9.3]
Liu 2017	1841	13705	13.4	[12.9; 14.0]
**Random effects model**		**80062**	**13.8**	**[11.0; 16.9]**
$I^2 = 100\%$, $\chi^2_4 = 875.78$ ($p < 0.01$)				

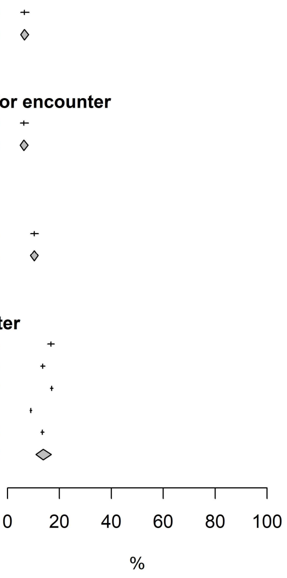

Figure 1: Meta-analysis: Prevalence/incidence of cough of all durations seen in primary care patients of all age groups in Asian, African and South American countries sorted by denominators. CI = confidence interval, k = number of (incidental) consultations because of a cough / patients in consultation for a cough / reason for encounter = cough, N = total number of (incidental) consultations / patients in consultation / reasons for encounter
