# Supplementary material for: Prevalence, aetiologies and prognosis of the symptom cough in primary care: a systematic review and meta-analysis
Source: BMC Fam Pract. 2021 Jul 12;22:151. doi: 10.1186/s12875-021-01501-0 (PMC8274469; doi:10.1186/s12875-021-01501-0)
Supplement: Supplementary file 4 — Additional file 4. Aetiologies of subacute and chronic cough. [file 12875_2021_1501_MOESM4_ESM.pdf]

Additional file 4: Aetiologies of subacute and chronic cough

Studies by aetiolgies	k	N	Percentage (%)	95% CI
**ACE-inhibitor induced**				
Nantha 2014	14	117	12.0	[6.9; 19.6]
not applicable				
**Acute bronchitis / bronchiolitis**				
Nantha 2014	11	117	9.4	[5.0; 16.6]
not applicable				
**Asthma**				
Munyati 2005	26	544	4.8	[3.2; 7.0]
Nantha 2014	5	117	4.3	[1.6; 10.2]
$I^2 = 0\%$, $\chi^2_1 = 0.01$ ($p = 0.92$)				
**COPD**				
Nantha 2014	8	117	6.8	[3.2; 13.4]
not applicable				
**Cryptococcosis**				
Munyati 2005	5	544	0.9	[0.3; 2.3]
not applicable				
**Fibrotic lung disease**				
Munyati 2005	34	544	6.2	[4.4; 8.7]
not applicable				
**GERD**				
Nantha 2014	7	117	6.0	[2.6; 12.4]
not applicable				
**Heart failure**				
Munyati 2005	15	544	2.8	[1.6; 4.6]
Nantha 2014	7	117	6.0	[2.6; 12.4]
$I^2 = 64\%$, $\chi^2_1 = 2.81$ ($p = 0.09$)				
**HIV-associated**				
Munyati 2005	454	544	83.5	[80.0; 86.4]
not applicable				
**Lower respiratory tract infection**				
Munyati 2005	178	544	32.7	[28.8; 36.9]
not applicable				
**Pertussis**				
Stefanoff 2014 *	288	3684	7.8	[7.0; 8.7]
not applicable				
**Pneumocystis pneumonia infection**				
Munyati 2005	8	544	1.5	[0.7; 3.0]
not applicable				
**Pneumonia**				
Munyati 2005	87	544	16.0	[13.1; 19.4]
Nantha 2014	3	117	2.6	[0.7; 7.9]
$I^2 = 95\%$, $\chi^2_1 = 22.2$ ($p < 0.01$)				
**Post infectious cough**				
Nantha 2014	42	117	35.9	[27.4; 45.3]
not applicable				
**Suspected malignancy**				
Munyati 2005	5	544	0.9	[0.3; 2.3]
Nantha 2014	2	117	1.7	[0.3; 6.7]
$I^2 = 0\%$, $\chi^2_1 = 0.78$ ($p = 0.38$)				
**Tuberculosis**				
Munyati 2005	234	544	43.0	[38.8; 47.3]
Nantha 2014	7	117	6.0	[2.6; 12.4]
$I^2 = 99\%$, $\chi^2_1 = 82.06$ ($p < 0.01$)				
**Upper airway cough syndrome**				
Nantha 2014	11	117	9.4	[5.0; 16.6]
not applicable				

Figure 1: Forest plot: prevalences of selected aetiologies, referring to adult primary care patients in consultation for a subacute or chronic cough, using the denominator patients. * = Stefanoff 2014 included patients of all age groups, ACE = Angiotensin-converting-enzyme, CI = confidence interval, COPD = Chronic obstructive pulmonary disease , GERD = Gastroesophageal reflux disease, k = number of patients with the respective aetiology, N = total number of patients in consultation for a cough.
